# Simulating hemispatial neglect with virtual reality

**DOI:** 10.1186/1743-0003-4-27

**Published:** 2007-07-19

**Authors:** Kenji Baheux, Makoto Yoshizawa, Yasuko Yoshida

**Affiliations:** 1 Graduate School of Engineering, Tohoku University, Sendai, Japan; 2 Information Synergy Center, Tohoku University, Sendai, Japan

## Abstract

**Background:**

Hemispatial neglect is a cognitive disorder defined as a lack of attention for stimuli contra-lateral to the brain lesion. The assessment is traditionally done with basic pencil and paper tests and the rehabilitation programs are generally not well adapted. We propose a virtual reality system featuring an eye-tracking device for a better characterization of the neglect that will lead to new rehabilitation techniques.

**Methods:**

This paper presents a comparison of eye-gaze patterns of healthy subjects, patients and healthy simulated patients on a virtual line bisection test. The task was also executed with a reduced visual field condition hoping that fewer stimuli would limit the neglect.

**Results:**

We found that patients and healthy simulated patients had similar eye-gaze patterns. However, while the reduced visual field condition had no effect on the healthy simulated patients, it actually had a negative impact on the patients. We discuss the reasons for these differences and how they relate to the limitations of the neglect simulation.

**Conclusion:**

We argue that with some improvements the technique could be used to determine the potential of new rehabilitation techniques and also help the rehabilitation staff or the patient's relatives to better understand the neglect condition.

## Background

Hemispatial neglect is a disorder usually observed after a stroke with a lesion in the right parietal lobe. It is defined as a loss of attention for stimuli contra-lateral to the lesion. In severe cases, patients with neglect can also suffer from associated disorders like hemi-paresis and somatoparaphrenia (regarding body parts as though they are someone else's) [1]. The assessment of hemispatial neglect is done with pencil and paper tests consisting of line bisection, target cancellation or copies of drawing tasks [2-4]. Patients with hemispatial neglect make incorrect bisections, fail to cancel the targets on the left side, etc. A system combining virtual reality and haptic feedback was developed to overcome the lack of proper quantification of neglect that occurs when using these traditional tests. The uniqueness of this system is the use of an eye-gaze tracking device.

Ishiai el al. [5] studied neglect patients for many years and in particular, has shown that eye-gaze pattern is a valuable information to gain an understanding of the underlying mechanisms of neglect. It can also be used to avoid a false positive result with hemianopia patients or as the basis of a quantitative assessment of neglect.

As we entered the testing phase, we had a lot of difficulties to find hemispatial neglect patients. This lead us to create a virtual equivalent of the neglect condition to artificially increase the number of patients. This paper explains how we simulate the neglect and presents a comparison study between the performance and eye-gaze patterns of patients, healthy subjects and healthy simulated patients on the virtual line bisection test.

## Methods

### Subjects

Two patients and 44 healthy subjects, shown in table 1, participated in the experiments. The healthy subjects were recruited among the student population and through a company specialized in the short-term employment for retired adults. Inclusion criteria included normal visual acuity (with or without correction), right hand dominance, the absence of any neuromuscular pathology. The healthy subjects were divided into a healthy subjects group and a healthy simulated patients group. Both groups were sub-divided into "young" and "senior" categories.

The two patients, Patient K, a male, and Patient O, a female, were recruited at the rehabilitation center of Saitou Hospital in Ishinomaki (prefecture of Miyagi, Japan). At the time of the experiment, Patient K. was 71 years old. On December 2005, he suddenly experienced difficulties to speak and went to a civic hospital where he was told he had a cerebral infarct. At the beginning of his rehabilitation in February 2006, he could not walk correctly because of the longer movements of his right foot. His unilateral spatial neglect condition was assessed on the basis of daily observations made by the rehabilitation staff. For instance, we were told that Patient K. usually bumps into various objects with his left shoulder or his left foot and often forgets to put on his left shoe. The hemispatial neglect was confirmed with line bisection and target cancellation pencil and paper tests prior to the experiment. Patient O., 73 years old, started her rehabilitation in March 2006. She had a cerebral infarct in January. The rehabilitation staff mentioned that she tends to not pay attention to her left hand. The assessment of the neglect with pencil and paper tests was not conclusive: a few missed or double checked targets and relatively fair results on the line bisection test. The eye-gaze pattern test was carried out in order to obtain additional information.

### Instruments

**Figure 1:**
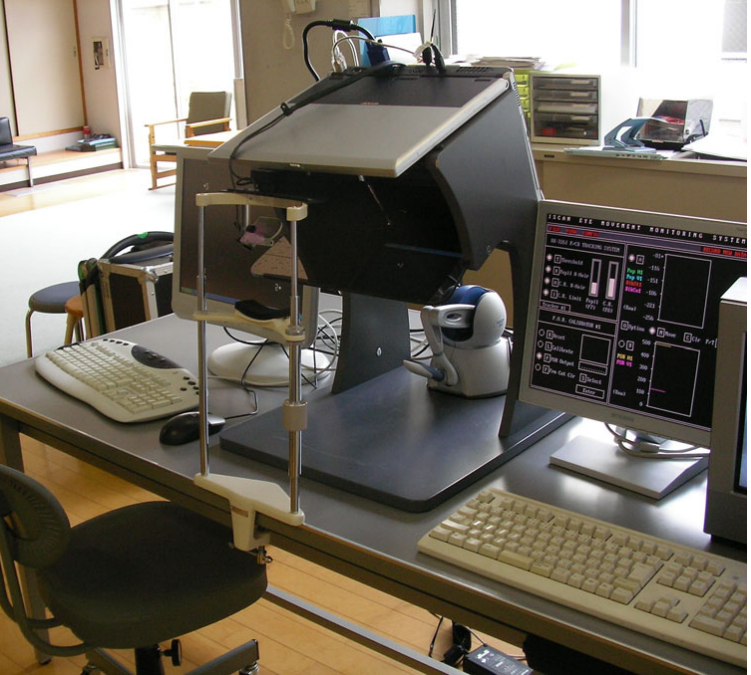
**System**. From left to right: virtual world monitoring, immersive workspace with eye-tracking device and a Phantom Omni haptics device, eye-tracking computer.

The system used in this study is shown in Fig. 1. It features an eye-tracking device, a haptic device and a Sharp Mebius PC-RD1-3D notebook. This notebook has a stereoscopic display that does not require the wearing of stereo glasses. A Phantom Omni made by Sensable, is used to interact with the virtual world. The notebook is mounted on a frame made by SenseGraphics in order to project the virtual world into the haptic space. The 3D-haptic world, including the audio environment, is processed by the notebook while a second IBM compatible personal computer controls the eye tracking device. The transmission of the current eye-gaze location is done through a serial link.

#### Tasks

This system was designed for the rehabilitation and assessment of patients with hemispatial neglect. Several of the tasks were specifically built for the assessment and other, more entertaining tasks for the rehabilitation. For the experiment presented in this paper, we used a virtual replica of the paper and pencil tests that was built to test new rehabilitation techniques and to provide a precise characterization of the neglect [6]. The assessment is based on the combination of performance and the eye-gaze pattern of the subject.

**Figure 2:**
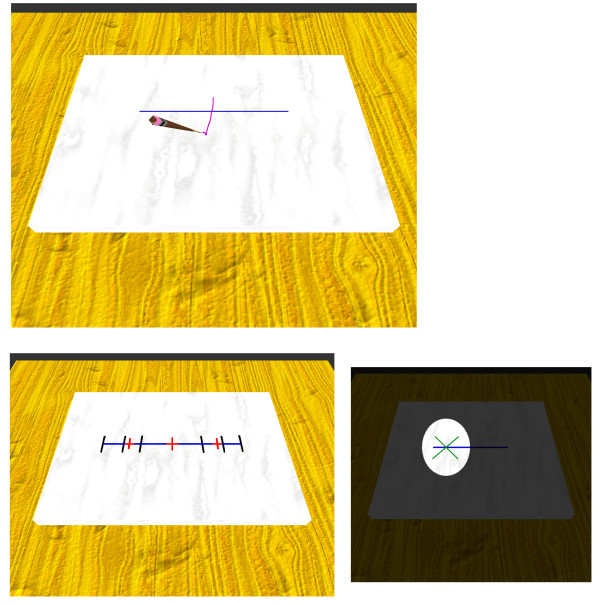
**Virtual line bisection**. Top: In the normal condition, the haptic device controls the pencil. When the pencil is lift up, the stroke is replaced by a cross if the stroke intersects the line. The subject can retry or validate his bisection and move on to the next trial. Bottom left: a montage showing the center of the lines (red crosses) in the three conditions (left sided, centered and right sided) and the extents of the lines when centered (in black). Bottom right: a montage showing the virtual line bisection in the reduced visual condition. The virtual world is viewed through a moving hole (only the bright area is visible). The subject has to stop the moving hole when the green cross reaches the middle of the line. When a choice is made, the whole virtual world is displayed. The subject can retry or validate and move on to the next trial.

For this experiment, we choose the well-known line bisection task, shown in Fig. 2. The virtual line bisection consists of marking the mid-point on 9 lines presented one at a time. The lines can have three different lengths (50 mm, 100 mm and 150 mm) and three different positions (left side, centered and right side). The trials were randomized and the origin of the haptic device was shifted by 25 mm to the right to avoid judgments based on the body midline. As an example of a new rehabilitation technique, we introduced a condition where the visual field is reduced in order to decrease the effect of the hemispatial neglect. The line bisection was performed in the normal condition and with a visual field reduced to a round area. This round area was constantly moving back and forth along the line.

As we had difficulties in finding many patients to test the system, we developed a simulation of the hemispatial neglect. The first computer model of hemispatial neglect was developed by Mozer [7] and is referred to as MORSEL. Our approach is simpler because we needed a real-time and interactive model. We use the eye-gaze tracking device to dynamically modify the virtual world to reflect the effect of the neglect. It was shown that the neglect can be object-centric or body-centric depending on the task [8,9]. In the case of the line bisection test, the neglect is a combination of both because evidence from other studies shows that the bisection of left sided lines has the poorest results and that the bisection of right sided lines is a bit better than that of centered lines [10,11]. However, given the small workspace and the fact that the patient's head and trunk are in a fixed position when using our system, we have chosen to implement an object-centric neglect. Only the right half of the line was displayed in the initial condition. The left part of the line was displayed depending on the eye-gaze. If the patient looks beyond the current left end of the line then the remaining segment between the eye-gaze and the current left end was displayed. In any case, the right half of the line was always displayed.

### Procedure

After a paper and pencil test evaluation of the hemispatial neglect, the subjects were told how to operate the system. For the normal condition, the subjects were told to "Mark the middle of the line with a pen stroke. The operator will then ask you if you want to try again. If you answer no, the operator will select the next trial.". For the reduced visual field condition, the instruction was "Press the button on the device when the green cross reaches the middle of the line. This will place a mark and display the whole line. The operator will ask you if you want to try again. If you answer no, we will move on to the next trial". The tasks were executed as follows : learning trial in the normal condition, one set in the normal condition, learning trial in the reduced visual field, one set in the reduced visual field, one set in the normal condition. The healthy simulated patients were not told about their particular condition and were given the same instructions.

### Analysis

The state of the virtual objects, the subject's interactions, eye-gaze and the positions of the marked mid-points were recorded. The analysis was done on the eye-gaze patterns and performance. Given the nature of the task, we only considered the lateral component of the eye-gaze. The subject's performance was evaluated by using the distance between the mark and the middle in terms of percents of half the length of the line. In other words, if the line was crossed at one of its extremities, the error would be 100%.

## Results

### Pattern of eye-gaze

**Figure 3:**
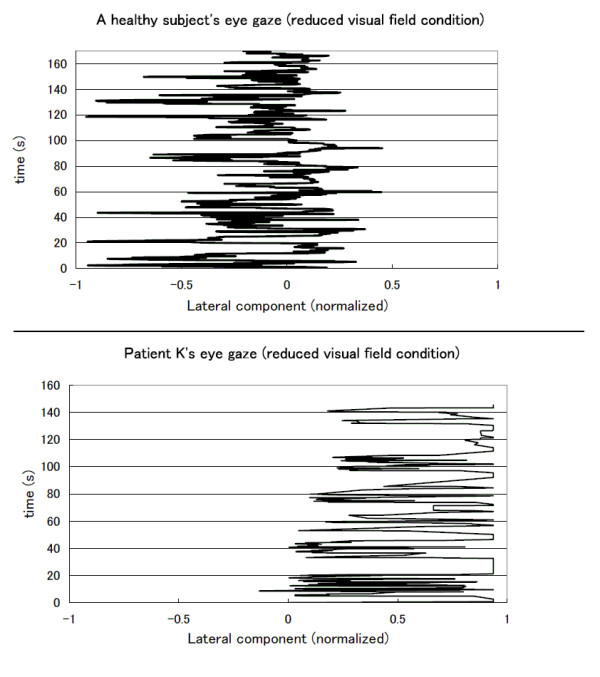
**Eye-gaze patterns**. Top: eye-gaze of a healthy subject during a task in the reduced visual field condition. Bottom: eye-gaze of Patient K. during a task in the reduced visual field condition.

We found that the eye-gaze pattern of the healthy subjects differed between the two conditions. In the normal condition, the eye-gaze was restricted to a central, narrow area occupying about half the width of the screen. For the reduced visual field condition, the upper graph of Fig. 3 shows a wider distribution that reaches the left end of the screen. We can also observe that the dominant area is still the middle of the screen. Similar patterns were observed for all the healthy subjects.

In contrast, there was no difference in the eye-gaze patterns of the healthy simulated patients for the two conditions. We observed a global shift of the distribution towards the left for all the simulated patients in both conditions. For the patient group, we found that the eye-gaze pattern of Patient O. covered a significant portion of the screen in both conditions. Patient K.'s eye-gaze patterns were constrained to the right side of the screen for the two conditions. The lower graph in Fig. 3 shows Patient K.'s eye-gaze during a reduced visual field task. The eye-gaze is clearly limited to the right half portion of the screen. The discontinuities are due to the unsuccessful tracking that happened when the patient's head was leaning toward the right side. However, we did not notice any peek to the left side while monitoring the video output of the eye-tracking device.

### Performance

**Figure 4:**
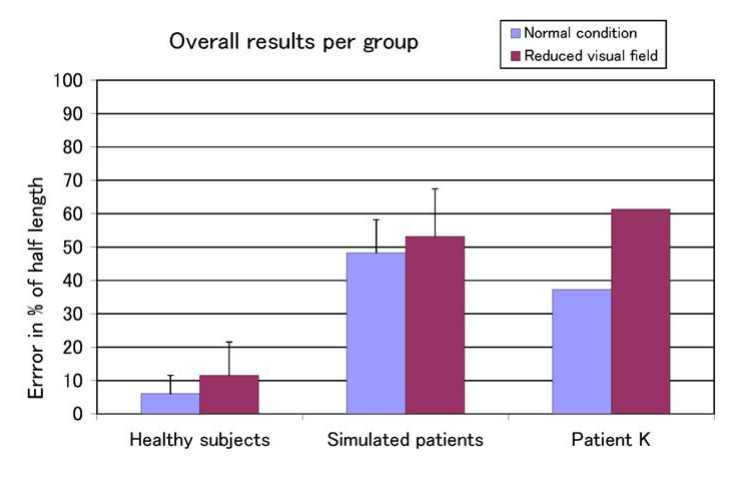
**Overall results of each groups for each condition**. The error represents the deviation from the true middle of the line. It is expressed in terms of the percentage of half the length of the line. An error of 100% means that the bisection was done on the extreme extent of the line.

We did not find any significant influence of the subjects' age over performance. Note that Patient O.'s results were excluded from this analysis. Her neglect seemed very light as her bisections were fair for a large number of trials. But more significant was the fact that her scanning pattern was not typical of neglect patients. Fig. 4 shows the average performance for the healthy subject, healthy simulated patients and for Patient K in the two conditions. The healthy subjects performed well in both conditions with a slight increase of the magnitude of the errors for the reduced visual field condition. The condition had no significant influence on the performance of the simulated patients. The slight increase of the magnitude of the errors was also present for the healthy simulated patients. Patient K.'s case was different as the condition had a significant (p < 0.05) effect on performance with an average error of 38% of half length in the normal condition to over 60% in the reduced visual field condition.

## Discussion

### Eye-gaze patterns

We were expecting that both the healthy simulated patients and patients would track the moving area in the reduced visual condition leading to better bisections and improvement for post-training tasks. Unfortunately, it was not the case. The healthy simulated patients had no reason to suspect that the lines were incomplete. Therefore, their strategy was to keep focusing on the middle of this line and wait for the return of the round area to place the mark. By comparison, the healthy subjects followed the round area to determine where the line would end. From there on, their strategy was similar to healthy simulated patients. Despite the similarities between the eye-gaze patterns of Patient K. and the healthy simulated patients, the implications are still unclear. For instance, the patients might have noticed that the line was not complete but could not follow the moving area because it entered their neglected space. Ishiai et al. reported that even if neglect patients are able to scan leftward when specifically asked to do so, their bisections do not improve significantly [12]. For our next experiments, we will specifically ask some of the patients to follow the round area beforehand to determine if the reduced visual field condition results in better bisections.

### Performance

**Figure 5:**
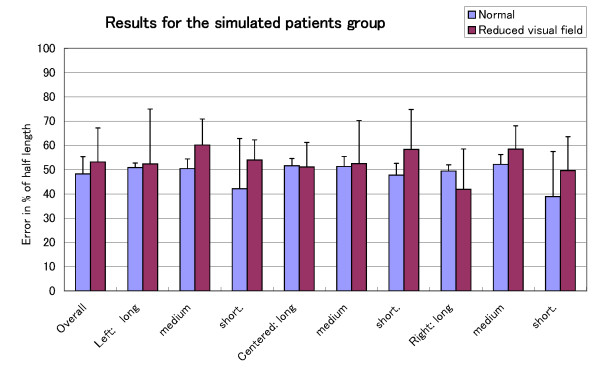
**Per trial results for the simulated patients group**. Performance of the simulated patients group in terms of the error in percent of half the length of the line for each trial ordered by location and length.

**Figure 6:**
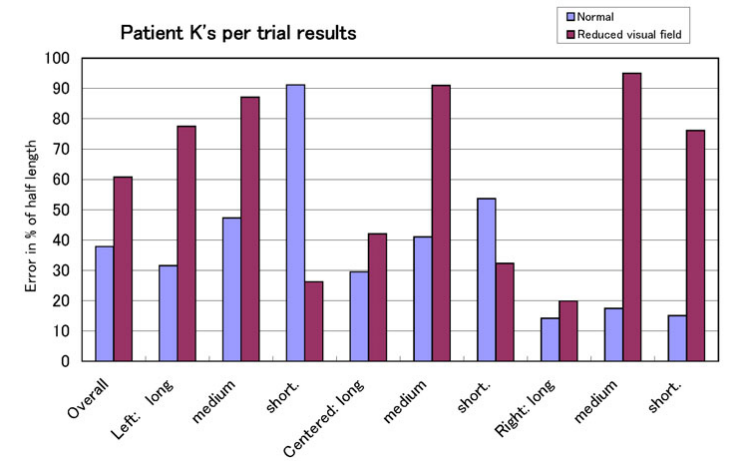
**Per trial results for Patient K**. Patient K.'s per trial performance for both conditions.

In Fig. 4, we can see that the performance in the reduced visual field condition is worse than in the normal condition for the three groups. However, while the condition had a significant effect on the performance of Patient K., this was not the case for the healthy simulated patients group. A closer look at the per trial performance of the healthy simulated patients group and Patient K. helps to explain the main reason behind this difference. Fig. 5 shows the per trial performance of the healthy simulated patients. Not only was there no effect of location on error, the average error for each trial was around 50%. This indicates that the lines were crossed as if they were half their length. The performance of Patient K. is shown in Fig. 6. The well-known effect of location on error can be observed in the normal condition but not in the reduced visual field condition where the decrease of performance is similar for all the trials. It may be that, in this particular condition, the bisection judgement is purely based on a mental image of the line for which the location of the line has no influence.

The difference with the patients' results shows the limit of our simulated hemispatial neglect which is based on the view introduced by Bisiach et al. [13] that neglect patients correctly bisect the rightward portion of the line that they see. We aim to adjust the algorithm to follow other models such as Marshall and Halligan's model based on scanning [14] or the view that neglect involves a distortion of the neglected side [15] and fine tune the model by using the patients' results.

## Conclusion

In this paper we have introduced the concept of simulating hemispatial neglect with virtual reality as a tool for the evaluation of new rehabilitation techniques. The present study indicates that this approach can be useful to determine the potential of a particular technique, such as reduction of the visual field, for intervention with hemineglect patients. Further experiments are needed to improve the simulation and to validate its use. Experiencing hemispatial neglect within a virtual reality system appears to benefit both the family and the rehabilitation staff. In order to meet this goal, the technique should be generalized to a wide variety of virtual scenes and extended to other sensory spaces.

## Competing interests

The author(s) declare that they have no competing interests.

## Authors' contributions

All authors read and approved the final manuscript. KB and YY were in charge of the software and hardware development, designed the tasks and experiments, conducted the analysis of the data and drafted the present paper. MY participated in the design of the study and helped to draft the manuscript.

**Table 1:** Subjects. Composition and details on the members of the different groups of subjects.

Groups	Sub-group	Male, Female	Mean age	Standard deviation
Healthy subjects	Young	11, 4	25.3	3.6
	Senior	2, 5	73.3	4.6
Simulated patients	Young	18, 2	23.15	2.0
	Senior	1, 1	70.5	9.2
Patients	N/A	1, 1	72.0	1.5
